# Human leukocyte antigen super-locus: nexus of genomic supergenes, SNPs, indels, transcripts, and haplotypes

**DOI:** 10.1038/s41439-022-00226-5

**Published:** 2022-12-21

**Authors:** Jerzy K. Kulski, Shingo Suzuki, Takashi Shiina

**Affiliations:** Department of Molecular Life Science, Tokai University School of Medicine, Isehara, Kanagawa Japan

**Keywords:** Haplotypes, Genetics research

## Abstract

The human Major Histocompatibility Complex (MHC) or Human Leukocyte Antigen (HLA) super-locus is a highly polymorphic genomic region that encodes more than 140 coding genes including the transplantation and immune regulatory molecules. It receives special attention for genetic investigation because of its important role in the regulation of innate and adaptive immune responses and its strong association with numerous infectious and/or autoimmune diseases. In recent years, MHC genotyping and haplotyping using Sanger sequencing and next-generation sequencing (NGS) methods have produced many hundreds of genomic sequences of the HLA super-locus for comparative studies of the genetic architecture and diversity between the same and different haplotypes. In this special issue on ‘The Current Landscape of HLA Genomics and Genetics’, we provide a short review of some of the recent analytical developments used to investigate the SNP polymorphisms, structural variants (indels), transcription and haplotypes of the HLA super-locus. This review highlights the importance of using reference cell-lines, population studies, and NGS methods to improve and update our understanding of the mechanisms, architectural structures and combinations of human MHC genomic alleles (SNPs and indels) that better define and characterise haplotypes and their association with various phenotypes and diseases.

## Immunity: Crucial cell surface proteins

New genetic sequencing and bioinformatic analysis techniques are improving our understanding of the human major histocompatibility complex (MHC), a large and complicated group of genes that encode cell surface proteins crucial to the human immune system. In a review, Jerzy Kulski and co-workers at the Tokai University School of Medicine in Isehara, Japan report that new sequencing technologies have now allowed researchers to map fine-scale and large-scale differences between different subgroups of genes within the MHC that are inherited together, called haplotypes. Study of haplotypes has illuminated the relatedness and evolutionary history of various human ethnic groups. Large-scale bioinformatic studies have revealed associations between MHC genes and disease. These advances will potentially provide better diagnosis or treatment for many diseases, both infectious and autoimmune, and may help improve transplant donor selection.

## Introduction

The human Major Histocompatibility Complex (MHC) on the short arm of chromosome 6 (band p21.3) is a Human Leukocyte Antigen (HLA) super-locus composed of clusters of many tightly linked supergenes involved with various phenotypic functions, mostly in connection with the immune response^1–4^. The MHC genes are defined as supergenes on the basis that they are clusters of tightly linked functional genetic elements spanning hundreds of kilobases that control complex balanced phenotypes and are inherited as a unit [haplotype] owing to reduced or absent recombination within them^5^, and because many have evolved by genomic duplications, deletions and inversions^6^. Although the most common mechanism of supergene formation is considered to be by inversion^7,8^, in which single crossovers between heterozygotes may lead to unbalanced gametes, the MHC genomic organisation reveals a variety of haplotypes with segmental duplications^9–11^, and structurally variant loci such as *C4* and *DRB*^12^, and a variety of duplicated repeat elements^6,13,14^, that exist possibly due to balancing selection^15,16^. These duplicated and inverted homologues probably generate recombinant haplotypes by varying rates of non-allelic and allelic homologous and nonhomologous recombinations and crossovers^12,17^. Thus, finding reliable phenotypic associations by genome-wide association studies (GWAS) is complicated and masked by the presence of hundreds of interlinked genes and regulatory elements in strong linkage disequilibrium (LD) within the super-locus^18–20^.

The HLA super-locus is characterised specifically by twelve classical class I and class II genes that encode antigen-presenting HLA proteins that present host (self) or foreign (nonself) peptides to interact with T-cell receptors in order to discriminate between self and nonself as part of the host immune response^3,20–23^. This is an important immunogenetic regulatory region^24^ of ~4 Mb in length with more than 120 non-HLA genes that together with the classical and non-classical HLA genes have been associated with more diseases than probably any other region of the human genome^1,2,12,25^. It is one of the most complex and diverse genomic regions with high levels of polymorphism, gene duplications, repeat elements, structural variations (indels), and long-range haplotype segments or blocks known as Conserved Extended Haplotypes (CEHs)^18^ or Ancestral Haplotypes (AHs)^10^. The diversity of the variable long-range haplotype segments within heterozygote individuals has provided problems and challenges for assigning SNPs to loci, and assembling structural variants of numerous duplicated genes particular in regard to associating them as genetic markers or causative agents for many of the immune-related phenotypes and diseases^18^. In recent years, more attention is being given to gaining a better understanding of MHC haplotypes by phased long-range sequencing as an extension of genotyping and identifying genic and non-genic alleles for associating them with disease, bone marrow transplantation, and for ascertaining the effects of immunotherapy^26^. Reliable MHC linkage mapping and haplotyping usually are dependent on pedigree studies of particular genotyped markers to evaluate their linkage or segregation in meiosis^18^ or on phased genomic sequences^26^, such as those that have been sequenced or genotyped using multilocus HLA-captured haplotype phasing^27,28^, *de novo* assembled trios^29^, MHC homozygous cell-lines^11^, sperm^30^ or single chromosomes^31^. Because of the complexity of the MHC as a HLA super-locus with a myriad of interconnected gene systems and sub-genomic regions, it is a gradual and continuing difficult process to build up the genetic, molecular and functional knowledge about the architectural and functional organisation of haplotypes in this region and their overall contribution to health and disease^1,2,25,26,32,33^.

In this brief review, we outline some of the recent analytical developments used to investigate the SNP polymorphisms, structural variants (indels), expression quantitative trait locus (eQTL) and haplotypes of the HLA super-locus. We highlight the importance of using reference cell-lines, population studies and next-generation sequencing (NGS) methods to overcome past problems and to improve and update our understanding of the mechanisms and architectural structures and combinations of human MHC genomic alleles (SNPs) that better define and characterise haplotypes, and their association with various phenotypes and diseases.

### MHC genomic sequence and subdivisions of structural organisation

**Fig. 1:**
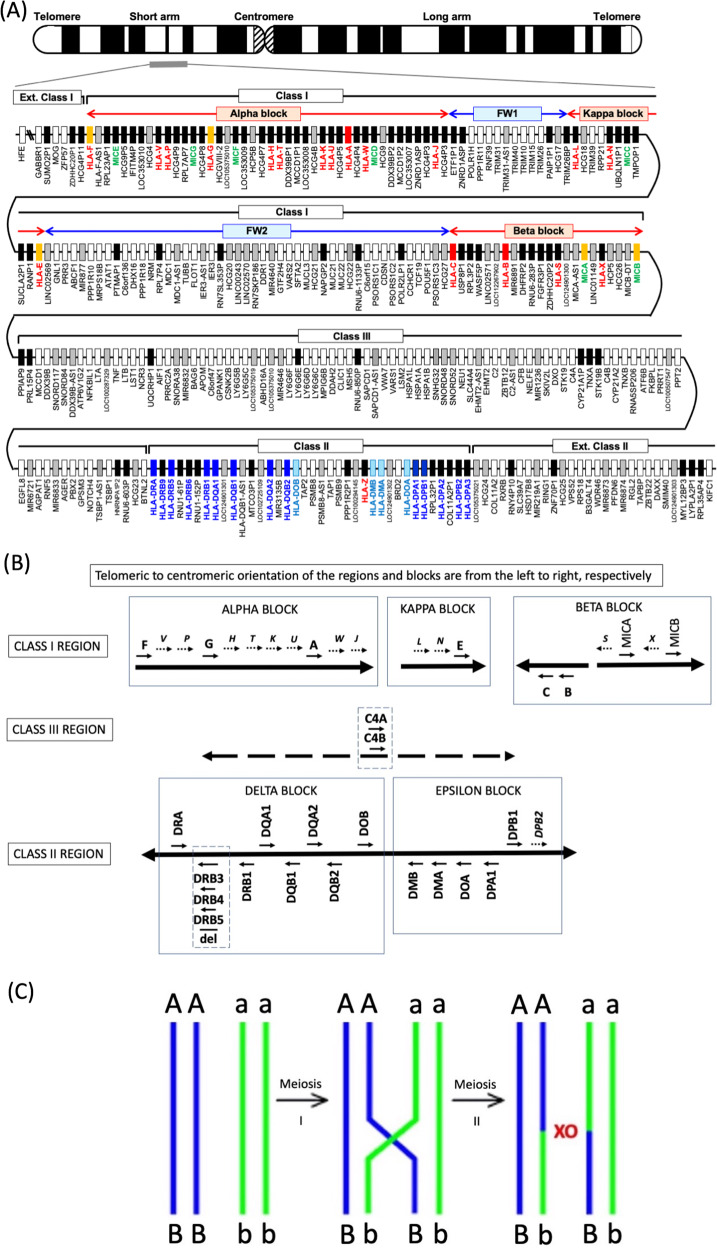
Human MHC genomic map, HLA gene duplications and haplotypic crossovers during meiosis. **A** Gene map of the HLA genomic region that corresponds to the genomic coordinates of 29602228 (*GABBR1*) to 33410226 (*KIFC1*) on chromosome 6 in the human genome GRCh38.p13 primary assembly of the NCBI map viewer. The regions separated by arrows show the HLA sub-regions such as extended class I, class I, class III, classical class II and extended class II regions from telomere (left and top side) to centromere (right and bottom side). The red and blue double, horizontal arrows show the spans of *alpha*, *kappa* and *beta* blocks, and framework (FW), non-HLA gene blocks, FW1 and FW2, respectively. The Class III region is composed of non-HLA genes or FW genes, but is known traditionally as Class III. White or coloured (orange, red and blue) boxes, grey, and black boxes show protein-coding genes, non-coding RNAs (ncRNAs), and pseudogenes, respectively. Red, green and blue letters indicate HLA class I, MIC, and class II genes, respectively. Adapted from Shiina et al.^1,2^. **B** *Cis* and *trans* structural orientation of duplicated and inverted HLA class I and class II genes within their duplication blocks (*alpha*, *kappa, beta, delta* and *epsilon*) relative to the telomeric and centromeric ends (left to right, respectively) of the HLA super-locus. The duplicated MIC pseudogenes, *MICE, MICG, MICF, MICD, MICC*, and their locations in the *alpha* block (**A**) are not shown in **B**. All MIC pseudogenes and genes in the MHC genomic region are coded in the opposite direction to all the HLA class I genes and pseudogenes^6^. Solid arrows indicate the 5′ to 3′ direction of coding genes and dotted arrows indicate the 5′ to 3′ direction of pseudogenes (italicised). The structural variants for the *HLA-DRB3, DRB4* and *DRB5* genes in the class II region and the *C4* genes in the class III region are indicated by the enclosed vertical boxes. The location and distribution of the duplicated genes are not shown to exact genomic scale. **C** Chromosomal or SNP density crossover (XO) junction in comparisons between two homozygous haplotype pairs (AB/AB and ab/ab) and two heterozygous recombinant (haplotype) pairs (AB/Ab and aB/ab). Chromosomal recombination is shown with a XO located between loci A and B within haplotype region ‘AB’ and between loci a and b within haplotype region ‘ab’ in a diploid cell during meiosis.

The first fully sequenced and gene annotated human genomic MHC was published in 1999 using the pioneering Sanger sequencing technology^34^. This primary sequence was a ‘virtual MHC’ composed of a mosaic of different human haplotypes rather than presenting any one particular haplotype. Subsequently, the first generation genomic sequences of eight human ancestral MHC haplotypes were published for a more precise comparative genomic analysis of the similarities and differences between different haplotypes^35^. Figure 1 shows the gene map of the HLA genomic region based on Genome Reference Consortium Human Build 38 patch release 14 (GRCh38.p14) in the National Center for Biotechnology Information (NCBI) database (https://www.ncbi.nlm.nih.gov/genome/?term=human) and the MHC-PGF haplotype, one of the eight MHC haplotypes sequenced by the MHC Haplotype Consortium (Fig. 1A)^35^. The MHC genomic organisation has a high degree of evolutionary complexity with the remnants of many homologous segmental duplications^6^ as well as inversions (Fig. 1B); probably turned over and shuffled by many different ancestral hominoid haplotypes as a result of non-allelic and allelic homologous recombination, gene conversion (nonhomologous recombination) and sequence crossover between different homozygotes or heterozygotes (Fig. 1C).

The HLA super-locus is divided into three regions related to the functions and distributions of the duplicated HLA genes and pseudogenes; the class I region located at the telomeric end and the class II region at the centromeric end, both separated from each other by an extended class III region of 61 protein-coding genes^1,2^. Whereas the HLA class I and class II genomic regions encode the highly polymorphic gene complex of the HLA class I and HLA class II genes, the class III region consists of many different non-HLA genes that are involved in stress response (*HSPA1A, HSPA1B and HSPA1L*), complement cascade (*C4A, C4B, C2, CFB*), immune regulation (*NFKBIL1, FXBPL* and *DDX39B*), inflammation (LTA, LTB, LST1, ABCF1, AIF1, NCR3 and TNF), leukocyte maturation (*LY6G5B, LY6GSC, LY6G6D, LY6G6E* and *LY6G6C*), and regulation of T cell development and differentiation (*BTNL2*)^4,36^. Recently, Zhou et al. showed that a quartet of MHC class III genes (*NELF-E, SKIV2L, DXO and STK19*) are involved with the metabolism and surveillance of RNA during the transcriptional and translational processes of gene expression^37^. The class II region also contains some proteosome-processing and peptide antigen transportation non-HLA genes such as *PSMB8*, *PSMB9, TAP1*, and *TAP2*. The TAP-binding protein, *TAPBP*, is in the extended class II region. The ‘Class I’ region (telomeric to centromeric ends) ranges from *HLA-F* to *MICB*, ‘Class III’ from *PPIAP9* to *BTNL2*, and ‘Class II’ from *HLA-DRA* to *HLA-DPA3*. There also are sub-regions from the telomeric side of Class I and the centromeric side of Class II that are called the ‘Extended class I’ (telomeric side of *HCG4P11*) and ‘Extended class II’ (centromeric side of *COL11A2*) regions, respectively. The class I region has been divided into three genomic blocks, *alpha*, *beta* and *kappa*^6,10,38^, that include duplicated HLA genes on either side of two intervening blocks of framework (FW1 and FW2) genes (Fig. 1A) that include non-HLA genes^39^. *HLA-A, -G* and *-F* are in the *alpha* block, *HLA-B* and *-C* are in the *beta* block, and *HLA-E* is in the *kappa* block.

A total of 283 loci were identified and/or reclassified in the 3.78-Mb HLA genomic region of the PGF haplotype from *GABBR1* located on the extended class I region to *KIFC1* located on the extended class II region (Fig. 1A and Table 1). When all the loci of the HLA genomic region are grouped into four categories of gene types, then 144 loci are classified as a protein-coding gene, 53 loci are non-coding RNA (ncRNA), five loci are small nucleolar RNA (snoRNA) and 81 loci are pseudogenes (Table 1). Of the 283 loci, 15.5% (44 loci) are occupied by HLA and HLA-like genes (HLA class I, HLA class II and MHC class I polypeptide-related sequences or MIC genes). However, the genic and non-genic numbers in Table 1 are not absolute for the MHC genomic region because of haplotype differences that may involve structural variations due to duplications, deletions, and insertions.

**Table 1:** Gene numbers in the HLA genomic region.

Gene status	Protein coding	ncRNA	snoRNA	Pseudo	Total
Extended Class I^a^	3	0	0	3	6
Class I	47	30	0	55	132
Class III	61	12	5	8	86
Class II	18	4	0	10	32
Extended Class II^b^	15	7	0	5	27
Total for all regions	144	53	5	81	283

^a^Extended class I is *GABBR1-HCG4P11*.

^b^Extended class II is *COL11A2-KIFC1*.

Of the HLA and HLA-like genes, 18 HLA class I genes (six protein-coding genes and 12 pseudogenes) (Fig. 1B) and 7 MIC genes (two protein-coding genes and five pseudogenes) are located in the HLA class I region, and 18 HLA class II genes (13 protein-coding genes and five pseudogenes) are in the HLA class II region (Fig. 1A and Table 2). Also, one HLA class I 88-bp pseudogene (*HLA-Z*) is located within the ncRNA gene *LOC100294145* close to the *HLA-DMB* gene in the HLA class II region. The classical HLA class I genes, *HLA-A, -B* and *-C*, and the classical HLA class II genes, *HLA-DR*, -*DQ* and -*DP*, are characterised by their extraordinary polymorphisms, whereas the non-classical HLA class I genes, *HLA-E*, *-F* and *-G*, are differentiated by their tissue-specific expression and limited polymorphism (Table 2).

**Table 2:** GRch38 MHC haplotype (PGF)s with HLA and MIC alleles, gene locations, and number of alleles at each gene locus.

HLA gene or pseudogene [P]	HLA-allele in GRch38	Genomic location Chr6, NCBI*	Gene ID	Number of alleles for each gene^a^
HLA-F	F*01:03:01:01	29,723,434–29,738,532	3134	59
HLA-V [P]	V*01:01:01:01	29,791,906–29,797,807	352,962	3
HLA-P [P]	P*02:01:01:02	29,800,044–29,803,079	352,963	5
HLA-G	G*01:01:01:05	29,826,474–29,831,021	3135	110
HLA-H [P]	H*02:04	29,887,573–29,891,079	3136	67
HLA-T [P]	T*03:01	29,896,443–29,898,947	352,964	8
HLA-K [P]	K*01:01:01:01	29,926,659–29,929,825	3138	6
HLA-U [P]	U*01:04	29,933,764–29,934,880	352,965	5
HLA-A	A*03:01:01:01	29,942,532–29,945,870	3105	7644
HLA-W [P]	W*01:01:01:05	29,955,834–29,959,058	352,966	11
HLA-J [P]	J*01:01:01:04	30,005,971–30,009,956	3137	33
HLA-L [P]	L*01:01:01:03	30,259,562–30,266,951	3139	5
HLA-N [P]	N*01:01:01:01	30,351,074–30,352,038	267,014	5
HLA-E	E*01:03:02:01	30,489,509–30,494,194	3133	342
HLA-C	C*07:02:01:03	31,268,749–31,272,092, comp	3107	7609
HLA-B	B*07:02:01:01	31,353,875–31,357,179, comp	3106	9097
HLA-S [P]	S*01:01:01:02	31,381,569–31,382,487	267,015	7
MICA	MICA*008:04	31,400,711–31,415,315	100,507,436	529
MICB	MICB*004:01:01	31,494,918–31,511,124	4277	237
HLA-DRA	DRA*01:02:03	32,439,887–32,445,046	3122	43
HLA-DRB5	DRB5*01:01:01:01	32,517,353–32,530,287, comp	3127	187
HLA-DRB1	DRB1*15:01:01:01	32,578,775–32,589,848, comp	3123	3389
HLA-DQA1	DQA1*01:02:01:01	32,637,406–32,655,272	3117	508
HLA-DQB1	DQB1*06:02:01:01	32,659,467–32,666,657, comp	3119	2330
HLA-DQA2	DQA2*01:01:01:03	32,741,391–32,747,198	3118	40
HLA-DQB2	DQB2*01:02:01	32,756,098–32,763,532, comp	3120	18
HLA-DOB	DOB*01:01:01	32,812,763–32,817,002, comp	3112	60
HLA-DMB	DMB*01:03:01	32,934,636–32,941,028, comp	3109	71
HLA-DMA	DMA*01:01:01	32,948,618–32,953,097, comp	3108	58
HLA-DOA	DOA*01:01:02	33,004,182–33,009,591, comp	3111	92
HLA-DPA1	DPA1*01:03:01:02	33,064,569–33,080,748	3113	491
HLA-DPB1	DPB1*04:01:01:01	33,075,990–33,089,696	3115	2221
HLA-DPA2	DPA2*01:01:01:01	33,091,482–33,093,314, comp	646,702	5
HLA-DPB2	DPB2*03:01:01:01	33,112,516–33,129,113	3116	6

^a^https://www.ebi.ac.uk/ipd/imgt/hla/about/statistics/ 17 October 2022.

^*^Assembly: GRch38p13 version, NC_000006.12 (https://www.ncbi.nlm.nih.gov/grc/human/regions/MHC?asm=GRCh38.p13).

Apart from the protein coding genes, pseudogenes, non-coding transcribed RNA loci, and small nucleolar transcribed RNAs (snoRNAs) loci, there are at least 8604 repeat elements including those known as transposable elements (TEs) and/or retroelements, and 723 simple repeats (microsatellites) in the MHC PGF haplotype sequence. Table 3 lists the main families of repeat elements identified and classified by RepeatMasker (http://www.repeatmasker.org) as a percentage of genomic sequence both within the intervening sub-regions, and within the entire MHC region from *HLA-F* to *HLA-DPA3*. The SINEs that congregated mainly in FW2 (26%) and class III (21%) regions were lowest in the *alpha, kappa, beta*, and class II blocks at <10%. The LINEs, mostly fragmented and of the mammalian L1M types, were found at highest percentage in the *kappa* block (31%), and within the *beta* block, FW1, and class II region, each at 26%. The ERVL subfamily of the LTR family were in the *alpha* and *beta* blocks at least at three to ten times higher percentage than within the other subregions. The LTR and ERVL were highest in the *alpha* block (25% and 13%, respectively) and lowest in the class III region (4% and 0.3%, respectively). Many of the LTR/HERVs form the building blocks of the transcriptional regulatory elements^40^, and their relatively high content in the *alpha* and *beta* blocks (Table 3) may reflect a role in the duplication of the HLA genes within the MHC^6,41–44^. The overall total percentage of the interspersed repeat elements (IREs) was highest in the *beta* (61%) and *alpha* (58%) blocks and lowest in the class III region (41%). On the other hand, the class III region and FW2 had the highest GC level percentage at 49% and 48%, respectively, possibly reflecting the greater density of coding genes within these two regions.

**Table 3:** Repeat elements as a percentage of genomic sequence within the intervening sub-regions and the entire MHC region from *HLA-F* to *HLA-DPA3*.

Block length bp	Alpha	FW1	Kappa	FW2	Beta	Class III	Class II	MHC all
	305,935	331,401	147,926	736,590	281,217	911,080	714,616	3,428,765
Repeat element (%)								
SINEs	6.61	12.54	9.01	26.24	7.56	21.75	9.88	16.26
* ALUs*	6.01	10.24	7.96	24.09	6.89	19.83	8.22	14.59
* MIRs*	0.60	2.30	1.05	2.11	0.63	1.92	1.65	1.66
LINEs	19.70	26.25	30.81	11.43	26.32	12.45	26.12	18.92
* LINE1 (L1*)	15.91	21.34	29.77	6.83	24.00	8.60	23.00	15.23
* LINE2 (L2)*	3.70	4.52	1.04	3.91	2.27	3.64	2.84	3.37
* L3/CR1*	0.09	0.21	0.00	0.32	0.05	0.21	0.24	0.20
LTR	24.77	9.36	12.48	11.88	23.05	4.02	14.05	12.16
* ERVL*	13.21	1.86	2.86	3.35	11.63	0.33	1.50	3.57
* ERVL-MaLRs*	7.03	4.93	4.83	2.09	2.66	0.63	3.25	2.84
* ERV-classI*	1.98	2.39	3.71	5.70	6.41	1.59	5.50	3.89
* ERV-classII*	2.57	0.00	1.08	0.41	2.36	1.39	3.62	1.68
DNA elements	5.50	4.60	3.23	2.18	1.89	1.84	4.01	3.00
* hAT-Charlie*	5.04	2.14	1.37	1.07	1.07	0.90	1.64	1.60
* TcMar-Tigger*	0.38	1.51	1.86	0.71	0.82	0.56	1.62	0.96
Unclassified	1.75	0.62	1.27	0.88	1.70	1.22	0.92	1.10
Total IR	58.33	53.36	56.80	52.61	60.52	41.28	54.97	51.44
Simple repeats (%)	1.28	0.67	0.99	0.81	1.06	0.98	0.80	0.92
GC level (%)	45.79	43.21	42.92	48.07	44.16	49.18	41.44	45.77

FW1 and FW2 indicate framework gene (non-HLA genes) segment 1 and segment 2, respectively, within the MHC class I region located between the *alpha* and *beta* blocks (Fig. 1A).

### Homozygous cell-lines as MHC genomic sequence haplotype references

Haplotypes at the genomic sequence level are blocks of phased coding and non-coding nucleotide sequences of multiple loci that are in the same orientation (*cis*) as their mode of gene transcription and regulation^26^. The characterisation and understanding of MHC haplotypes in modern disease and population genetics began in 1967 with the introduction of the word *‘haplotype’* by Ruggero Ceppellini to describe alleles in the HLA system^45^, and expanded in the 1990s with the pedigree studies of the research groups of Alper^9,18^, and Dawkins^10,46,47^. Since then, the International Histocompatibility Workshop Group (IHWG) has provided at least a thousand commercially available cell-line samples from HLA heterozygous and homozygous donors, families, and diverse populations (https://www.fredhutch.org/en/research/institutes-networks-ircs/international-histocompatibility-working-group.html) that are important for research into MHC immunogenetics, comparative genomics, transcriptomics and haplomics^11,18,28,35,46,47^ These genotyped or fully sequenced MHC haplotypes provide standardised references to assist with the design and interpretation of HLA genotyped population studies and HLA-disease relationships. The genotyped cell-lines also provide excellent insights into the structural organisation of MHC phased haplotypes^11^, not previously available for detailed comparative analysis by just using blood or tissues samples collected from diploid heterozygous individuals. The first MHC genomic sequence variations in different haplotypes were produced by the Sanger Centre MHC Haplotype Project (SCMHP) using eight homozygous cell-lines^35^. These now are alternative reference sequences as part of the human reference genome GRCh38^48^. Initially, only two haplotypes were resolved completely at the base pair level (cell-lines PGF and COX); whereas the other six haplotypes were completed only at 51% (cell-line APD) to 93% (cell-line QBL) of the MHC genomic region. Seven of the SCMHP cell-lines were resequenced again as part of 95 near-complete haplotypes, using short-range and long-range NGS^11,49^. Overall, Norman et al. provided 137 genotyped loci for most of the 95 cell-lines that they sequenced^11^.

Table 4 shows the diversity of 68 different haplotypes at six HLA class I and class II loci for eight cell-lines sequenced by the SCMHP, and 82 IHWG reference cell-lines sequenced, genotyped, and annotated by Norman et al.^11^ whereas Norman et al.^11^ genotyped for polymorphisms at 139 MHC loci in the MHC class I, II and III regions, for simplicity, the haplotypes listed in Table 4 are shown only for the six HLA class I and class II loci of the classical genes, *HLA-A, -C, -B, -DRB1, -DQA1* and *-DQB1*. Nevertheless, these 68 examples illustrate the segmental organisation of the haplotypes, whereby some blocks of consecutive loci are (1) the same or highly similar (homozygous, conserved, shared or matched), (2) different (heterozygous or diverse), or (3) a hybrid recombinant (mixed) composed of adjoining blocks of conserved and different sequences^12–14,50^. The AH/CEH nomenclature in Table 4 is taken from Dorak et al.^47^. The AH names use the B allele and if two or more AH carry the same B allele then sequential numbers are added to indicated the order of discovery, such as AH7.1 and AH7.2^47^. In Table 4, four different cell-lines (PGF, SCHU, HO104, LD2B)^11^ have the haplotypic structure of AH7.1^47^, which is a ‘homozygous’ or ‘conserved’ haplotype represented by the HLA lineage alleles A*03-C*07-B*07-DRB1*15-DQA1*01:02-DQB1*06. AH7.2 has C*07-B*07, but differs to AH7.1 at A*24-C*07-B*07-DRB1*01-DQA1*01:01-DQB1*05^47^. Similarly, AH8.1^47^ is highly conserved in five different homozygous cell-lines (COX, STEINLIN, VAVY, L0541265, PF04015) with the HLA lineage alleles of A*01-C*07-B*08-DRB1*03-DQA1*05-DQB1*02 at six loci. These haplotype nomenclatures can be expanded from the one allelic set of digits up to four or six sets of digits. For example, the following AH8.1^47^ is classified using 4 allelic digital numbers at five HLA loci: A*01:01-C*07:01-B*08:01-DRB1*03:01-DQA1*05:01-DQB1*02:01.

**Table 4:** Diversity of different haplotypes at six HLA class I and class II loci.

HLA-A	HLA-C	HLA-B	HLA-DRB1	HLA-DQA1	HLA-DQB1	No. cells	AH
(A) MHC Haplotype Project (Horton et al.^35^)							
A*01:01:01	C*06:02:01	B*40:01:01	DRB1*13:01:01	DQA1*01:03:01	DQB1*06:03:01	APD	60.x
A*01:01:01	C*07:01:01	B*08:01:01	DRB1*03:01:01	DQA1*05:01:01	DQB1*02:01:01	COX	8.1
A*02:01:01	C*03:04:01	B*15:01:01	DRB1*04:01:01	DQA1*03:03:01	DQB1*03:01:01	MCF	62.2
A*02:01:01	C*06:02:01	B*57:01:01	DRB1*07:01:01	DQA1*02:01:01	DQB1*03:03:02	DBB	57.1
A*03:01:01	C*07:02:01	B*07:02:01	DRB1*15:01:01	DQA1*01:02:01	DQB1*06:02:01	PGF	7.1
A*26:01:01	C*05:01:01	B*18:01:01	DRB1*03:01:01	DQA1*05:01:01	DQB1*02:01:01	QBL	18.2
A*29:02:01	C*16:01:01	B*44:03:01	DRB1*07:01:01	DQA1*02:01:01	DQB1*02:02:01	MANN	44.2/44.3
A*32:01:01	C*05:01:01	B*44:02:01	DRB1*04:03:01	DQA1*03:01:01	DQB1*03:05:01	SSTO	44.x
(B) Norman et al. (2017) Haplotype Project^11^							
A*01:01:01	C*01:21	B*52:01:01	DRB1*15:02:01	DQA1*01:03:01	DQB1*06:01:01	1	52.x
A*01:01:01	C*03:03:01	B*15:01:01	DRB1*13:01:01	DQA1*01:03:01	DQB1*06:03:01	1	62
A*01:01:01	C*04:01:01	B*35:02:01	DRB1*11:02:01	DQA1*05:05:01	DQB1*03:01:01	1	35.5
A*01:01:01	C*04:01:01	B*35:02:01	DRB1*11:04:01	DQA1*01:03:01	DQB1*06:03:01	1	35.x
A*01:01:01	C*06:02:01	B*37:01:01	DRB1*16:01:01	DQA1*01:02:02	DQB1*05:02:01	1	–
A*01:01:01	C*06:02:01	B*40:01:02	DRB1*13:01:01	DQA1*01:03:01	DQB1*06:03:01	1	60.x
A*01:01:01	C*06:02:01	B*57:01:01	het	het	het	1	–
A*01:01:01	C*07:01:01	B*08:01:01	DRB1*03:01:01	DQA1*05:01:01	DQB1*02:01:01	5	8.1
A*01:01:01	C*07:01:01	B*49:01:01	DRB1*11:02:01	DQA1*05:05:01	DQB1*03:19	1	–
A*01:01:01	C*17:01:01	B*41:01:01	DRB1*11:01:01	DQA1*05:05:01	DQB1*03:01:01	1	–
A*02:01:01	C*01:02:01	B*27:05	DRB1*01:01:01	DQA1*01:01:01	DQB1*05:01:01	1	–
A*02:01:01	C*02:02:02	B*27:05:02	DRB1*16:01:01	DQA1*01:02:02	DQB1*05:02:01	1	–
A*02:01:01	C*02:02:02	B*40:02:01	DRB1*16:01:01	DQA1*01:02:02	DQB1*05:02:01	1	60.x
A*02:01:01	C*03:04:01	B*15:01:01	DRB1*04:01:01	DQA1*03:01:01	DQB1*03:02:01	1	62.1
A*02:01:01	C*04:01:01	B*35:01:01	DRB1*08:01:01	DQA1*04:01:01	DQB1*04:01:01	1	35.x
A*02:01:01	C*05:01:01	B*44:02:01	DRB1*11:01:01	DQA1*01:02:02	DQB1*05:02:01	1	44.x
A*02:01:01	C*05:01:01	B*44:02:01	DRB1*04:01:01	DQA1*03:03:01	DQB1*03:01:01	1	44.1
A*02:01:01	C*05:01:01	B*44:02:01	DRB1*14:54:01	DQA1*01:04:01	DQB1*05:03:01	1	44.x
A*02:01:01	C*06:02:01	B*57:01:01	DRB1*07:01:01	DQA1*02:01	DQB1*03:03:02	2	57.1
A*02:01:01	C*07:01:01	B*57:01:01	DRB1*16:02:01	DQA1*01:02:02	DQB1*05:02:01	1	57.x
A*02:01:01	C*12:03:01	B*35:03:01	het	het	het	1	–
A*02:01:01	C*01:02:01	B*27:05:02	DRB1*08:01:01	DQA1*04:01:01	DQB1*04:01:01	1	–
A*02:01:01	C*03:04:01	B*15:01:01	DRB1*04:01:01	DQA1*03:03:01	DQB1*03:01:01	2	62.x
A*02:01:01	C*03:04:01	B*40:01:02	DRB1*08:01:01	DQA1*04:01:01	DQB1*04:02:01	1	60.2
A*02:01:01	C*03:04:01	B*40:01:02	DRB1*13:02:01	DQA1*01:02:01	DQB1*06:04:01	1	60.3
A*02:01:01	C*05:01:01	B*18:01:01	DRB1*11:02:01	DQA1*05:05:01	DQB1*03:01:01	1	18.x
A*02:01:01	C*07:01:01	B*18:01:01	DRB1*12:01:01	DQA1*05:05:01	DQB1*03:01:01	1	18.x
A*02:01:01	C*07:01:01	B*18:01:01	DRB1*14:54:01	DQA1*01:04:01	DQB1*05:03:01	1	18.x
A*02:01:01	C*12:03:01	B*38:01:01	DRB1*13:01:01	DQA1*01:03:01	DQB1*06:03:01	1	38.x
A*02:01:01	C*16:01:01	B*45:01:01	DRB1*13:01:01	DQA1*01:03:01	DQB1*06:03:01	1	–
A*02:01:01	C*06:02:01	B*13:02:01	DRB1*07:01:01	DQA1*02:01	DQB1*02:02:01	1	13.1
A*02:04	C*15:02:01	B*51:01:01	DRB1*16:02:01	DQA1*05:05:01	DQB1*03:01:01	2	51.x
A*02:05:01	C*07:18:01	B*58:01:01	DRB1*03:01:01	DQA1*05:01:01	DQB1*02:01:01	1	58.x
A*02:12	C*01:02:01	B*51:01:01	DRB1*08:01:01	DQA1*04:01:01	DQB1*04:02:01	1	51.x
A*02:17:02	C*03:03:01	B*15:01:01	DRB1*03:02:01	DQA1*05:03	DQB1*03:01:01	2	62.x
A*03:01:01	C*06:02:01	B*50:01:01	DRB1*07:01:01	DQA1*02:01	het	1	50.x
A*03:01:01	C*07:02:01	B*07:02:01	DRB1*04:01:01	DQA1*03:01:01	DQB1*03:02:01	1	7.3
A*03:01:01	C*07:02:01	B*07:02:01	DRB1*15:01:01	DQA1*01:02:01	DQB1*06:02:01	4	7.1
A*11:01:01	C*04:01:01	B*35:01:01	DRB1*01:01:01	DQA1*01:01:01	DQB1*05:01:01	1	35.2
A*11:01:01	C*04:01:01	B*35:03:01	DRB1*14:04	DQA1*01:04:02	DQB1*06:01:01	1	35.x
A*23:01:01	C*05:01:01	B*14:01:01	DRB1*04:01:01	DQA1*03:01:01	DQB1*03:02:01	1	–
A*24:02:01	C*01:02:01	B*54:01:01	DRB1*04:01:01	DQA1*03:03:01	DQB1*04:01:01	1	54.1
A*24:02:01	C*03:04:01	B*40:01:02	DRB1*09:01:02	DQA1*03:02	DQB1*04:01:01	1	60.x
A*24:02:01	C*04:01:01	B*15:01:01	DRB1*04:06:01	DQA1*03:01:01	DQB1*04:01:01	1	62.x
A*24:02:01	C*04:01:01	B*35:08:01	DRB1*11:03	DQA1*05:05:01	DQB1*03:01:01	1	35.4
A*24:02:01	C*01:02:01	B*56:01	DRB1*16:01:01	DQA1*01:02:02	DQB1*05:02:01	1	–
A*24:02:01	C*12:02:02	B*52:01:01	DRB1*15:02:01	DQA1*01:03:01	DQB1*06:01:01	2	52.1
A*24:02:01	C*12:03:01	B*51:01:01	DRB1*01:01:01	DQA1*01:01:01	DQB1*05:01:01	1	51.x
A*24:02:01	C*07:02:01	B*07:02:01	DRB1*01:01:01	DQA1*01:01:01	DQB1*05:01:01	1	7.2
A*26:01:01	C*05:01:01	B*18:01:01	DRB1*03:01:01	DQA1*05:01:01	DQB1*02:01:01	1	18.2
A*26:01:01	C*12:03:01	B*38:01:01	DRB1*04:02:01	DQA1*03:01:01	DQB1*03:02:01	1	38.1
A*26:01:01	C*07:01:01	B*08:01:01	DRB1*15:01:01	DQA1*01:02:01	DQB1*06:02:01	1	8.x
A*29:02:01	C*16:01:01	B*44:03:01	DRB1*04:01:01	DQA1*03:03:01	DQB1*03:01:01	1	44.x
A*29:02:01	C*16:01:01	B*44:03:01	DRB1*07:01:01	DQA1*02:01	DQB1*02:02:01	2	44.2
A*30:01:01	C*06:02:01	B*13:02:01	DRB1*07:01:01	DQA1*02:01	DQB1*02:02:01	1	13.x
A*30:02:01	C*05:01:01	B*18:01:01	DRB1*03:01:01	DQA1*05:01:01	DQB1*02:01:01	2	18.x
A*31:01:02	C*01:02:30	B*15:01:01	DRB1*08:02:01	DQA1*04:01:01	DQB1*04:02:01	1	62.x
A*31:01:02	C*15:02:01	B*51:01:01	DRB1*04:07:01	DQA1*03:03:01	DQB1*03:01:01	1	51.x
A*31:01:02	C*03:04:01	B*40:01:02	DRB1*04:04:01	DQA1*03:01:01	DQB1*03:02:01	1	60.1
A*31:01:02	C*04:01:01	B*35:01:01	DRB1*04:01:01	DQA1*03:03:01	DQB1*03:01:01	1	35.x
A*32:01:01	C*05:01:01	B*44:02:01	DRB1*13:02:01	DQA1*01:02:01	DQB1*06:04:01	1	44.x
A*32:01:01	C*05:01:01	B*44:02:01	DRB1*04:03:01	DQA1*03:01:01	DQB1*03:05:01	1	44.x
A*32:01:01	C*12:03:01	B*38:01:01	DRB1*11:01:01	DQA1*05:05:01	DQB1*03:01:01	1	38.x
A*33:01:01	C*08:02:01	B*14:01:01	DRB1*01:02:01	DQA1*01:01:02	DQB1*05:01:01	1	65.1
A*33:01:01	C*08:02:01	B*14:01:01	DRB1*07:01:01	DQA1*02:01	DQB1*02:02:01	1	64.x
A*33:03:01	C*14:03	B*44:03:01	DRB1*13:02:01	DQA1*01:02:01	DQB1*06:04:01	1	44.4
A*66:01:01	C*12:03:01	B*38:01:01	DRB1*14:01:01	DQA1*01:04:01	DQB1*05:03:01	1	38.x
A*68:02:01	C*04:01:01	B*53:01:01	DRB1*15:03:01	DQA1*01:02:01	DQB1*06:02:01	1	–
					Total	82	–

The haplotypes in (A) and (B) were sorted according to the HLA-A allele in descending order. The AH nomenclature is taken from Dorak et al.^47^, which is based on the initial definitions by Dawkins et al.^10^ and Alper et al.^9,18^, whereby the AHs are also called CEHs. The AHs are named using the B allele, and if two or more AHs carry the same B allele then sequential numbers are added to indicate their order of discovery, such as AH7.1 and AH7.2. The ‘x’ after the B allele implies that the sequential number is not known, and therefore needs to be updated. A blank space in the AH column indicates that the AH designation is not known or updated in the literature. Norman et al.^11^ have provided the names of the cell-lines for each of the haplotypes sequenced, but we have not added them to this table for brevity, and prefer to indicate the number of different cell-lines that were sequenced with the same HLA class I and class II alleles.

**Fig. 2:**
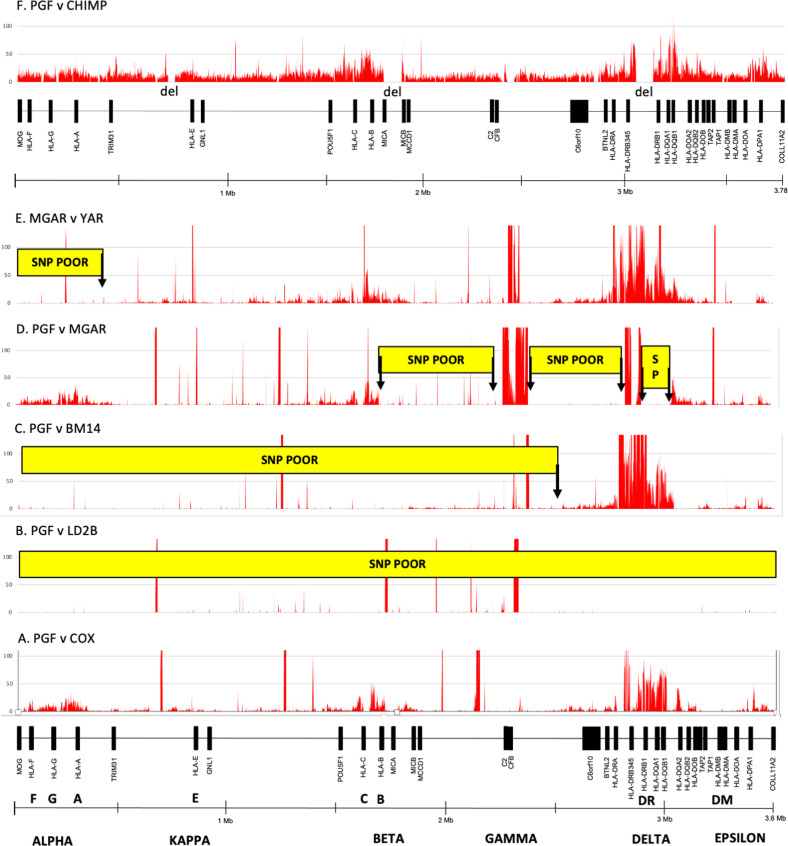
SNP or SNV density plots between different paired alignments of MHC haplotypes represented by six homozygous cell-lines, PGF, COX, LD2B, BM14, MGAR, YAR and a chimpanzee (CHIMP) genomic reference sequence, GCF_002880755.1 (Clint_PTRv2). The MHC gene markers and genomic distances (Mb) from left to right between the *MOG* and *COL11A2* genes, and the regions of polymorphic frozen blocks known as *alpha, kappa, beta, gamma, delta* and *epsilon* (Dawkins et al.^10^, Shiina et al.^2^), are shown at the bottom of the Figure. The four SNP plots (**A**–**D**) are between the haplotype PGF: A*03:01-C*07:02-B*07:02-DRB1*15:01-DQA1*01:02-DQB1*06:02 and the haplotypes of **A** COX: A*01:01-C*07:01-B*08:01-DRB1*03:01-DQA1*05:01-DQB1*02:01:01, **B** LD2B: A*03:01-C*07:02-B*07:02-DRB1*15:01-DQA1*01:02-DQB1*06:02, **C** BM14: A*03:01-C*07:02-B*07:02-DRB1*04:01-DQA1*03:01-DQB1*03:02 and **D** MGAR: A*26:01-C*07:01-B*08:01-DRB1*15:01-DQA1*01:02-DQB1*06:02. The fifth SNP plot (**E**) is between the haplotype MGAR (see D) and YAR: A*26:01-C*12:03-B*38:01-DRB1*04:02-DQA1*03:01-DQB1*03:02. In **F**, the SNV plot is between the PGF reference sequence (CZUC02000001.1) and the chimpanzee (CHIMP) genomic reference sequence, GCF_002880755.1. The SNV regions label ‘del’ are genomic sequence regions absent from CHIMP sequence. The Chimpanzee *MIC* gene in the beta block is a hybrid of human *MICA* and *MICB*^53,62^. The Y-axis presents the number of SNP/kb (window size). The X-axis shows the SNP density positions (SNP/kb) across 3.6 Mb of genomic sequence between the *MOG* and *COL11A2* genes. The red vertical lines along the X-axis that are above 100 on the Y axis are artifactual sequences or those representing sequence gaps, poor assembly, inversions or long runs of unspecified nucleotides. The yellow horizontal boxes labelled SNP POOR are regions of recombination (highly conserved nucleotide sequence with little or no SNPs between sequence alignments). In this context, the SNP POOR regions are those that are <1 SNP/kb, in contrast to the same regions that are SNP rich (>1 SNP/kb) in other haplotype sequence comparisons. The MHC class III and most FW genes in the class I region are always SNP poor, and consequently were not labelled as such in **A**, **D** or **E**. The ends of the ‘SNP POOR’ boxes represent regions of putative crossovers (vertical arrows) between SNP poor and SNP rich regions of different haplotypes in **C**–**E**. In B, PGF v LD2B shows the relative absence of SNPs across 3.6 Mb between two conserved (highly similar sequences) haplotypes. Extended genomic regions (>50 kb) with 1-50 SNP/kb are considered to be SNP rich regions, whereas extended regions of < 1 SNP/kb are SNP poor regions. The SNPs within SNP poor regions were easy to count manually because of small numbers (<0.1 SNP/kb), whereas SNP rich regions were difficult to count because of larger numbers at an average of 7 SNP/kb in the alpha block (320 kb), and up to 50 SNP/kb or greater in the delta block (185 kb) depending on haplotype comparisons. In the alpha block, the highest average SNP density between seven different haplotypes was 16 SNP/kb near *HLA-A* with the lowest density at ~2 SNP/kb near the *HLA-J* pseudogene^13^. The SNP count was <0.001 SNP/kb between the same *HLA-A* haplotypes in **C** and **D**. Spikes and peaks of SNPs above 100 SNP/kb were due mostly to nucleotide misalignments because of poor sequence assembly, structural variations, gaps, inversions or long runs of unspecified nucleotides. See recent SNP plots by Houwaart et al.^49^ for additional comparisons between MHC haplotypes.

The allelic combinations of the BOLETH cell-line (AH62.1) and the MCF cell-line (A*02-C*03-B*15-DRB1*04-DQA1*03-DQB1*03) are totally different to those of the AH7.1 and AH8.1 cell-lines at the six MHC loci. The AH7.1 and AH8.1 allele lineages^47^ are different from each other at all the six loci except at *HLA-C* where they are both C*07; although they actually are different from each other at the two digital allelic level, C*07:02 and C*07:01, respectively. This two digital allelic difference represents the two amino acid difference between the HLA-C proteins for AH7.1 (PGF) and AH8.1 (COX) with K90N in exon 2 and S125Y in exon 3. Comparatively, most of the 68 haplotypes in the Norman et al.^11^ study are hybrids or recombinants that are different at one or more loci, but share the same alleles possibly at other loci. For example, the ten haplotypes with the allele A*01:01:01:01 at the *HLA-A* locus are different at one or more of the other five loci. However, some of these A*01 haplotypes have the same alleles at other loci. There are two haplotypes that are both A*01:01:01-C*07:01:01, but different from each other at the *HLA-B, -DRB1, -DQA1* and *-DQB1* loci. Similarly, there are two haplotypes that both have A*01:01:01-DRB1*11:01/02:01-DQA1*05:05:01, but differ from each other at the *HLA-C* and *-B* loci. This illustrates the considerable mixing and matching between different haplotypes in a process called shuffling^50,51^. Similarly, trends of loci shuffling are evident for the 21 haplotypes with A*02:01:01:01, and so on. Genomic sequence comparisons between MHC class I or between class II ‘hybrid’ haplotypes by Kulski et al.^13,14^ suggest that the haplotypic block or segmental SNP patterns with genomic sequence crossovers (Fig. 2) probably evolved ancestrally using recombination mechanisms^17^. Conserved and hybrid haplotypes are likely to have accumulated in interrelated populations or ethnic groups in relatively recent times, possibly over a few thousand generations or more^52^. These shuffling or recombination mechanisms are delineated also as SNP diversity plots in sequence alignments between two phased MHC genomic regions (Fig. 2).

#### Haplotype SNP diversity plots and crossover junctions

Figure 2 shows SNP diversity plots in nucleotide DNA comparisons between the same and different human MHC haplotypes as well as to that of a chimpanzee haplotype sequence. SNPs are the nucleotide sequence differences seen between two different phased haplotypes that have been aligned (Fig. 2A, E, F). Sequence alignments between different haplotypes (heterozygous sequences) reveal varying SNP densities (number of SNPs per kb) across the entire MHC with the greatest SNP densities occurring in the *alpha* block within the *HLA-A* gene region; the *HLA-B* and *-C* genes of the *beta* block; the *delta* block with *HLA-DRB1*, -*DQA1* and -*DQB1*; and the *epsilon* block involving *HLA-DPB1*. Unsurprisingly, the highest SNP density peaks occur in the regions of the HLA classical class I and class II genes that correlate positively with the overall number of alleles detected for the different HLA gene loci (Table 2). In comparison, the SNP densities are consistently at low levels in the non-HLA genetic regions such as those between the *alpha* and *beta* blocks in the class I region, and in the class III region where the number of alleles for each of the class III genes are often <20, and comparable to the allele numbers detected for non-classical HLA genes, like *HLA-F*, and HLA pseudogenes (Table 2).

Fewer SNPs are detected between two aligned homologous or highly similar sequences (e.g., Fig. 2B, PGF versus LD2B) than between different haplotypes (e.g., Fig. 2A, PGF v COX) because they are identical by descent with no recombination. However, some nucleotide differences either as *de novo* mutations and/or sequencing or assembly errors are evident across the alignment between fully matched HLA loci (conserved haplotypes). In contrast, sequence alignments of recombinant haplotypes (e.g., Fig. 2C–E) reveal an extended sequence block that is rich in SNPs adjoining an extended block of homologous sequences with no or few SNPs (labelled as a SNP poor or SP) that are seen to be SNP rich in other haplotype comparisons (Fig. 2A). The junction between the SNP rich and SNP poor blocks are the SNP crossover junctions suggesting that they are in close proximity to chromosomal recombination crossover regions^13,14^, as outlined in Fig. 1C. With recombinations and crossovers, a considerable amount of opportunistic hitchhiking may occur particularly near the HLA loci^53^, and with the integration and rearrangement of Alu, LTR and HERV elements^54^.

#### Supergene expression, eQTL, epistasis and disease

Since undertaking our earlier analyses of MHC gene variants, epistatic interactions, expression activity and associations with various diseases taken from publications and records in public databases such as the Gene Expression Omnibus (GEO), Online Mendelian Inheritance in Man (OMIM) and the Genetic Association Database (GAD)^1,2^, these types of genome-wide MHC association studies have progressed much further with the more formidable bioinformatic analyses of phenotype associations, known as MHC PheWAS^55^. However, regulatory elements can act over long distances and in a cell-type specific manner that hamper the easy identification of the causal genes for a given pathological condition^56,57^. In this regard, haplotyped homozygous cell-lines also can be used to study gene interactions or epistasis both inside and outside the MHC genomic region^16,58,59^. Expression quantitative trait locus (eQTL) studies associate genomic and transcriptomic data sets from the same individuals to identify loci that affect mRNA expression by linking SNPs to changes in gene expression^58^. Thus, eQTL analysis can be an useful procedure for annotating GWAS variants.

A number of recent studies using homozygous cell-lines and/or biological samples have demonstrated that the expression of various clusters of genes inside or outside the MHC genomic region can be affected by the expression of one or more haplotypic genes within the MHC genomic region^58–61^. Lam et al. used eight homozygous cell-lines, six with Chinese haplotypes (A*33:03-C*03:02-B*58:01-DRB1*03:01 or A*02:07-C*01:02-B*46:01-DRB1*09:01), and two with European haplotypes (A*01:01-C*07:01-B*08:01-DRB1*03:01)^58^. They used haplotypic RNA and DNA-sequencing data to show that haplotype sequence variations represented by eQTL SNP alleles can function as *cis*-acting regulatory variants for multiple MHC genes. The enriched haplotype-specific transcriptional eQTLs were localised especially within four segmental regions containing *HLA-A* (*alpha* block), *HLA-C* (*beta* block), *C4A* (*gamma* block) and *HLA-DRB* (*delta* block). Thirty-six MHC genes from extended MHC and classes I, II and III showed significantly differential expression between the three MHC haplotypes.

Lamontagne et al. used hundreds of lung tissue samples collected from patients in Canada and the Netherlands to show that gene expression within the extended MHC region and class I, II and III regions correlated with lung disease/trait specific local- and distant-acting eQTL SNPs^60^. By using eQTL analysis of a large human cohort with both RNA-sequencing and genotyping data available for HLA alleles in peripheral blood, Sharon et al. found strong trans-regulatory associations between the HLA-DR, HLA-DQ, or HLA-DP β chains and the T cell receptor (TCR) α chains^61^. Their results suggest that MHC genotypes have a key role in shaping the TCR repertoire by determining the *V* gene usage profiles of an individual’s TCR repertoire. In a recent in-depth interrogation of associations between genetic variation, gene expression and disease, D’Antonio et al. showed that eQTL analyses of HLA haplotypes provided substantially greater statistical power than only using single variants^59^. They examined the association between AH8.1 and delayed colonisation in Cystic Fibrosis, and suggested that downregulation of *RNF5* expression was the likely causal mechanism. Taken together, these pioneering eQTL studies incorporating HLA haplotypes are a powerful approach to identify causal genetic mechanisms underlying disease associations both inside and outside the MHC region. In this regard, we recently developed a new RNA-sequencing method to capture differential allele-level expression and genotypes of all the classical HLA loci and haplotypes in the Japanese population for further in-depth studies of graft rejection after transplantation and HLA-related diseases^28^.

#### Structural variants: indels and transposable elements in MHC genomic evolution and regulation of expression

**Fig. 3:**
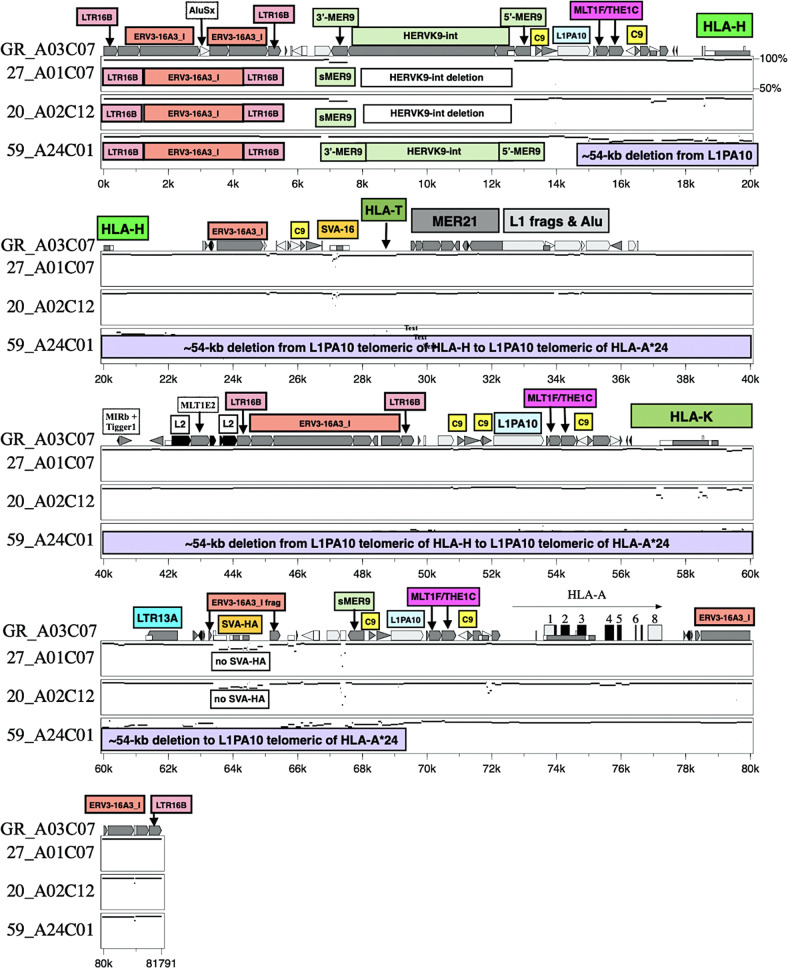
Genomic map with identity plots of a 54-kb deletion (purple box) between *HLA-G* and *HLA-A* in the 59_HLA24C01 haplotype sequence compared to the aligned sequences of the GR_HLA-A03C07, 27_A01C07 and 20_A02C12 haplotypes listed on the left side of the figure. The locations of *HLA-H*, *HLA-T*, *HLA-K* pseudogenes (labelled green boxes) *HLA-A* (horizontal arrow) and some TE are indicated on the GR_A03C07 sequence. The yellow box labelled C9 represents *Charlie9*. The location of the intact telomeric *HLA-G* gene and the deleted pseudogene *HLA-U* centromeric of *HLA-K* are not shown. All interspersed repeats in the upper sequence are indicated with the symbols used by Kulski et al.^13^.

The human MHC structural variants and indels have received far less attention than SNPs and minor variants with respect to health and disease. In comparative genomic analyses between different MHC haplotypes, the indel diversity is two to seven times greater than SNP diversity^53,62^. Structural variants and indels have a potential gain and loss of functions that can affect phenotypes, susceptibility and resistance to disease *via* many different molecular, cellular and pathogenic independent and interrelated mechanisms. Figure 3 shows an ~55-kb deletion within the alpha block of a haplotype with HLA-A*24:02^13^ that has the highest allele frequency of 35.6% in the Japanese population (http://hla.or.jp/med/frequency_search/en/allele/). HLA-A*24:02:01 apparently has a protective effect against Stevens-Johnson syndrome (SJS) and toxic epidermal necrolysis (TEN) that are life-threatening acute inflammatory vesiculobullous reactions of the skin and mucous membranes^63^.

Transposable elements (TEs) have important, albeit, often poorly defined roles in generating haplotypes via recombination mechanisms such as integration (insertion), duplication, rearrangements, deletions and gene conversion^64,65^. TEs and other repeat sequences appear to have been integral in the generation of MHC segmental duplications of the class I and class II regions^6,66^, and of different haplotypes, mainly by acting both as recombination acceptor and suppression sequence regions for DNA binding Rec proteins and enzymes such as PRDM9 depending on their genomic distribution, sequence conservation or diversity, and evolutionary age of integration and transposition^13,14^. The association of particular TEs and repeats with MHC segmental duplications were reported previously for the genomic structural organisation of MHC duplicated genes in humans^6^, chimpanzees^38,62^ and rhesus macaques^67^. Both old and young Alu insertions generate point mutations, microsatellites and SNPs within the flanking regions of the insertion sites^68^. TEs such as Alu, SVA, HERVs and LTR have been used as genetic markers to estimate the evolutionary age of MHC gene duplication events and for discerning the evolutionary interrelationships between different human haplotypes^54,66,69^. For example, ten young AluY indels that are either present or absent in particular human MHC class I and class II haplotypes are useful evolutionary genetic markers of past recombination events, as well as excellent markers for elucidating population phylogenetics and genetic interrelationships^70–72^. In this regard, Cun et al. recently showed that five different MHC class II dimorphic Alu elements either alone or linked together as haplotypes with *HLA-DRB1* alleles can differentiate 12 Chinese minority ethnic groups according to their geographic locations, and correlate them with their population characteristics of language family, migration and sociality^73^.

TE insertions within the MHC genomic region might act like surgical sutures or band-aids that help to repair and rejoin double-strand DNA breaks during recombination events^41^, such as those involved with the ‘mismatch repair system’ or via various other repair mechanisms of damaged DNA^17^. In this regard, it seems that TEs like Alu, L1, SVA and LTR are involved intimately with recombination, DNA repair, as well as contributing to nucleotide point mutations between different sequences^6,13,41^. Moreover, some of these TE indels have been strongly associated with the regulation of gene expression and disease^74,75^. Much work is needed to characterise which MHC TEs have contributed to past recombination events, affect gene expression, and have a role in MHC related diseases, and various important traits and phenotypes associated with pathogen defence.

### Population MHC haplotypes

Although homozygous cell-lines can provide phased genomic sequences for analysis of haplotypic structures, population studies are necessary for information about the frequency and distribution of the MHC haplotypes and their association with disease, and for obtaining cross-matching data for organ and cell transplantations. Most frequency data of population MHC haplotypes are based on genotyping HLA alleles of heterozygotes and applying statistical and computation methods such as the expectation-maximisation algorithm or LD values of non-random, multi-allelic correlations between pairs of loci to estimate the correct phase of the haplotypes^76^. The LD statistical analysis of heterozygotes might be reasonably accurate for estimating high frequency or common haplotypes, but the reliability decreases for low frequency or minor haplotypes. Confounders to haplotype estimations include typing ambiguity, sample size, incompleteness of HLA data, allele frequency errors, recombination and especially unknown gamete phase.

A number of family-based population studies were published in the 1980s and 1990s on extended MHC haplotype frequencies for Caucasians in Australia^77^, and the United States^78^, as well as for American non-dominant European Caucasian and non-Caucasian or admixed Caucasian/non-Caucasians^18^. Since then, the HLA haplotype frequencies have been determined for many more different worldwide populations^79,80^, and ethnic groups using pedigrees or statistical inference (http://www.allelefrequencies.net/default.asp). Table 5 lists examples of the six most common HLA haplotype frequencies for Japanese, Chinese, Saudi, British Caucasians, European Americans (Caucasians) and African Americans deduced by LD inference or segregation by pedigree analysis. Although we used the British Caucasian population as an example of the common European haplotypes such as AH7.1, AH8.1 and AH44.1 (Table 5), the European HLA haplotype frequencies vary markedly among European populations across the European continent^80^. According to Dawkins and Lloyd^46^, the five most common MHC AH haplotypes (at five HLA loci) in Australian Europeans living in Perth, Western Australia are AH8.1 (13.2%), AH7.1 (12.9%), AH44.1 (5.5%), AH44.2 (2.6%) and AH57.1 (2.6%), frequencies which tend to reveal a large immigratory bias towards their British ancestors (Table 5).

**Table 5:** Six most common HLA haplotype frequencies in six world populations.

Population and HLA haplotypes (some with CEH/AH designations)	Freq (%)
Japanese, 768 families, 3072 haplotypes (Shiina et al., unpublished data)	
A*2402-C*1202-B*5201-DRB1*1502-DQA1*0103-DQB1*0601-DPA1*0201-DPB1*0901	7.3
A*2402-C*0702-B*0702-DRB1*0101-DQA1*0101-DQB1*0501-DPA1*0103-DPB1*0402	3.2
A*3303-C*1403-B*4403-DRB1*1302-DQA1*0102-DQB1*0604-DPA1*0103-DPB1*0401	3.1
A*2402-C*0102-B*5401-DRB1*0405-DQA1*0303-DQB1*0401-DPA1*0202-DPB1*0501	2.0
A*1101-C*0401-B*1501-DRB1*0406-DQA1*0301-DQB1*0302-DPA1*0103-DPB1*0201	1.2
A*0207-C*0102-B*4601-DRB1*0803-DQA1*0103-DQB1*0601-DPA1*0202-DPB1*0202	0.9
Chinese, 8608 segregated haplotypes (Li et al.^95^)	
A*3001-C*0602-B*1302-DRB1*0701-DQB1*0202	5.0
A*0207-C*0102-B*4601-DRB1*0901-DQB1*0303	3.2
A*3303-C*0302-B*5801-DRB1*0301-DQB1*0201	2.8
A*3303-C*0302-B*5801-DRB1*1302-DQB1*0609	1.5
A*1101-C*0801-B*1502-DRB1*1202-DQB1*0301	1.3
A*0207-C*0102-B*4601-DRB1*0803-DQB1*0601	0.9
Saudi, 3,588 LD inferred haplotypes (Jawdat et al.^96^)	
A*0201-C*1502-B*5101-DRB1*0402-DQB1*0302-DPB1*0401	1.0
A*0201-C*0702-B*0702-DRB1*1501-DQB1*0602-DPB1*0401	0.9
A*0201-C*0602-B*5001-DRB1*0701-DQB1*0201-DPB1*0401	0.8
A*2301-C*0602-B*5001-DRB1*0701-DQB1*0201-DPB1*0301	0.6
A*2402-C*0702-B*0801-DRB1*0301-DQB1*0201-DPB1*0401	0.6
A*0101-C*1701-B*4101-DRB1*0701-DQB1*0303-DPB1*0402	0.6
British Caucasian, 11,088 PHASE imputed haplotypes (Neville et al.^97^)	
A*0101-C*0701-B*0801-DRB1*0301-DQA1*0501-DQB1*0201 (AH8.1)	7.5
A*0301-C*0702-B*0702-DRB1*1501-DQA1*0102-DQB1*0602 (AH7.1)	3.0
A*0201-C*0501-B*4402-DRB1*0401-DQA1*0301-DQB1*0301 (AH44.1)	2.6
A*0201-C*0702-B*0702-DRB1*1501-DQA1*0102-DQB1*0602 (AH7.x)	1.8
A*2902-C*1601-B*4403-DRB1*0701-DQA1*0201-DQB1*0202 (AH44.2)	1.8
A*0101-C*0602-B*5701-DRB1*0701-DQA1*0201-DQB1*0303 (AH57.x)	1.4
European American, 12768 statistically inferred haplotypes (Maiers et al.^98^)	
A*0101-C*0701-B*0801-DRB1*0301-DQB1*0201 (AH8.1)	7.4
A*0301-C*0702-B*0702-DRB1*1501-DQB1*0602 (AH7.1)	3.5
A*0201-C*0501-B*4402-DRB1*0401-DQB1*0301 (AH44.1)	2.4
A*0201-C*0702-B*0702-DRB1*1501-DQB1*0602 (AH7.x)	2.3
A*2902-C*1601-B*4403-DRB1*0701-DQB1*0201 g (AH44.2)	1.8
A*0101-C*0602-B*5701-DRB1*0701-DQB1*0303 (AH57.x)	1.3
African American, 894 statistically inferred haplotypes (Maiers et al.^98^)	
A*3001-C*1701-B*4201-DRB1*0302-DQB1*0402 (AH42.1)	1.5
A*0101-C*0701-B*0801-DRB1*0301-DQB1*0201 (AH8.1)	1.4
A*0301-C*0702-B*0702-DRB1*1501-DQB1*0602 (AH7.1)	0.9
A*3303-C*0401-B*5301-DRB1*0804-DQB1*0301	0.8
A*6802-C*0304-B*1510-DRB1*0301-DQB1*0201	0.7
A*6801-C*0602-B* 5802-DRB1*1201-DQB1*0501 (AH58.x)	0.7

The AH nomenclature is taken from Dorak et al.^47^. The ‘x’ after the AH B allele is an unknown sequential number that needs to be updated.

The conserved or fixed haplotypes that have little diversity and no evidence of recombination within their genomic sequences such as AH7.1 or AH8.1 of Caucasian individuals (Table 5) can be studied and described as ‘identity by descent’ (IBD) haplotypes^81^, which are distinct from ‘identity by state’ (IBS) haplotypes, that is, those that have emerged by convergence. The highly conserved haplotypes that are shared between generations (haplotype sharing) might remain fixed or frozen over long periods of evolutionary time because of founder effects and population bottlenecks^82^, as well as efficient DNA repair mechanisms, negative population selection, or as yet unknown mutation inhibitory mechanisms. To what degree are conserved haplotypes frozen or fixed? Although this question is not resolved fully, available data suggest that many inherited haplotypes are not completely identical and that *de novo* mutations, SNPs and/or indels, in MHC genomic sequence comparisons do exist between the same conserved haplotypes^83–86^. The identification of variants between the same haplotypes might have importance in assisting with optimal donor-recipient selection for allogeneic stem cell transplantation and with reducing acute and chronic graft-versus-host disease^26^.

On the other hand, heterozygous haplotypes or those that are very different between individuals (e.g., AH7.1 and AH8.1) are likely to have been inherited by an interplay of various genetic and population evolutionary processes including recombination, positive selection of benign mutations or SNPs, gene flow, genetic drift, frequency-dependent selection, admixture and trans-speciation over long periods of evolution^15,16,80^. For example, the known MHC class I haplotype sequences of Japanese, Africans, Asians, Arabs and Europeans generally are all different to each other in phylogenetic analyses^86,87^. Despite haplotype sharing of high frequency conserved polymorphic sequences by IBD such as those for AH8.1 or AH7.1^10,52^, most haplotypes among Europeans and other populations (Table 5) generally are markedly different in structure, organisation and frequency as a consequence of various hypothetical genetic and population evolutionary processes^80^.

### Conclusion: third generation sequencing

The new knowledge gathered during the past decade on the architectural complexity and diversity of MHC haplotype genomic sequences stems largely from DNA and RNA sequencing methods, but remains incomplete because it is difficult to assign SNPs correctly to loci and assemble structural variants of numerous duplicated genes within individuals by using the first generation Sanger sequencing method or the short read NGS technology^88,89^. Despite the large number of genomes produced by second generation sequencing, their quality is compromised by the relatively short reads (usually <250 bp) used to construct them (typically from Illumina sequencing by synthesis)^89^. Long-read sequencing by third generation sequencing (TGS) together with the many improved bioinformatic tools allow the longer regions of genomic sequence with repetitive elements to be assembled for more reliable haplotype reconstruction^90–94^. Pacific Biosystems (PacBio) and Oxford Nanopore can generate reads over 10 kb^91^, which makes TGS ideal for assembling genomes in areas with gene duplications^27,28^, repetitive elements^90^ and for generating long haplotype blocks^91–93^. Thus, TGS along with pan-genome bioinformatic analyses have the potential to better assist with haplotype phasing, and for elucidating haplotype regulatory modules within the HLA super-locus and their association with a wide range of complex diseases, including infectious and autoimmune diseases.
